# Application of OFA-based ERAS for video-assisted thoracoscopic surgery in elderly patients with airway stenosis: A case report

**DOI:** 10.1097/MD.0000000000037662

**Published:** 2024-04-19

**Authors:** Mengya Yang, Danmin Wang, Xia Xu, Xiaobo Yu, Hefei Xu, Zhaoqiang Zeng, Jingwei Dai

**Affiliations:** a Department of Anesthesiology, People’s Hospital of Wanning, Wanning Hainan China; b Department of Anesthesiology, The First Affiliated Hospital of Hainan Medical University, Haikou, Hainan China; c Department of Anesthesiology, Sanya Central Hospital, Sanya, Hainan China; d Department of Neurosurgery, People’s Hospital of Wanning, Wanning Hainan China; e Department of Thoracic surgery, People’s Hospital of Wanning, Wanning Hainan China.

**Keywords:** enhanced recovery after surgery, esketamine, opioid-free anesthesia, spontaneous breathing, tubeless anesthesia, video-assisted thoracoscopic surgery

## Abstract

**Background:**

Thoracic surgery without general anesthesia can be traced back to the First World War, and thoracic epidural block was used to complete the operation due to a large number of patients with gunshot wounds who needed emergency thoracic surgery. By reducing the intraoperative opioid dose, intraoperative and postoperative opioid-related adverse events such as respiratory depression, nausea and vomiting, delirium, hyperalgesia, and other side effects can be reduced to the benefit of patients.

**Methods:**

A 72-year-old male patient was admitted to the hospital with a 5-day history of multifocal pain throughout the body caused by a fall. The injury was not treated at that time, and the pain gradually increased, accompanied by cough with difficulty expelling sputum.

**Diagnoses:**

Left lung contusion; traumatic pneumonia; multiple left rib fractures; left fluid pneumothorax; thyroid tumor of unknown nature, possibly malignant. Grade I tracheal stenosis; Sequelae of cerebral infarction. Because of goiter and severe tracheal compression, the patient was not intubated and received deopiated general anesthesia combined with epidural anesthesia to preserve spontaneous breathing.

**Outcomes:**

At the end of the video-assisted thoracoscopic exploration, the patient was immediately conscious and returned directly to the ward 6 min later. The patient was able to move freely after surgery and eat normally within 6 h of surgery. The postoperative visual analog scale score was 2 points, and there were no anesthetic complications during the follow-up.

**Conclusion:**

The opioid-free anesthesia strategy of tubeless general anesthesia, allowing spontaneous breathing combined with epidural anesthesia in elderly patients with tracheal stenosis undergoing video-assisted thoracoscopic surgery can not only avoid accidents and injuries caused by tracheal intubation and mechanical ventilation, but can also significantly reduce postoperative respiratory complications, optimize postoperative analgesia, and help achieve enhanced recovery after surgery.

## 1. Introduction

Tubeless general anesthesia can reduce the incidence of complications such as intubation-related airway injury, residual neuromuscular block, ventilation-induced lung injury, cardiac dysfunction and postoperative nausea. In the 1950s, Buckingham and Vichniewsky reported more than 600 cases of thoracic epidural anesthesia with preserved spontaneous breathing for thoracic surgery.^[1]^ With the later emergence of the double-lumen endotracheal tube, endotracheal intubation general anesthesia quickly became the gold standard for thoracic surgery, and non-endotracheal intubation thoracic anesthesia was gradually forgotten. With the development of modern medical technology and the improvement of medical techniques, especially the emergence of thoracoscopic technology in surgery, as well as the development and progress of anesthesia monitoring, airway management, anesthetic drugs and anesthesia theory, thoracic anesthesia with preserved spontaneous breathing can return to clinical practice in an updated form. At present, a series of related reports have been published by a number of research centers in China and abroad, which believe that tubeless anesthesia is safe and feasible, and helps to achieve rapid recovery after surgery. Perioperative opioids offer a good analgesic effect, but can also lead to nausea and vomiting, respiratory depression, delirium, hyperalgesia, and other side effects,^[2–4]^ thereby prolonging hospital stay and delaying recovery. Several retrospective studies have also demonstrated the feasibility and effectiveness of opioid-free anesthesia (OFA) in thoracic surgery, lowering morphine consumption while reducing postoperative pain scores.^[5,6]^ As an adjuvant drug, esketamine has the advantages of maintaining airway tension and hemodynamic stability, which makes it an ideal choice for anesthesia and sedation.^[7]^ Based on these developments, enhanced recovery after surgery programs have been successfully implemented in thoracic surgery.^[8–10]^

## 2. Case presentation

The patient (male, 72 yrs old, 172 cm, 63 kg, ASA grade III) had a painful left chest injury for 5 days, caused by a fall while walking. The pain was accompanied by dizziness and headache, but he had no chest tightness, shortness of breath, dyspnea, cough or sputum on initial presentation. No treatment was given at the time, but the pain gradually worsened, accompanied by cough with difficulty expelling sputum, so he was admitted to our hospital. He had a past history of “sequelae of cerebral infarction.” He denied a history of surgery, anesthesia, or medication.

### 2.1. Physical examination

**Figure 1.**
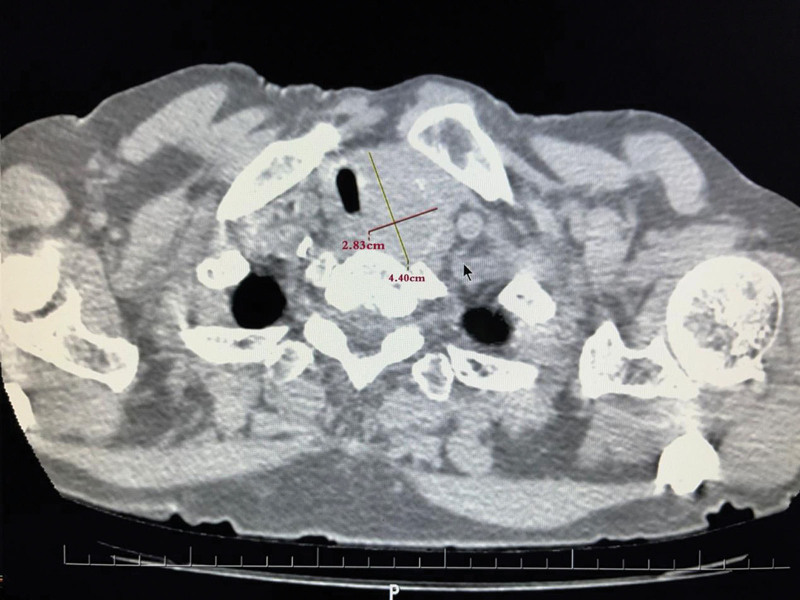
The patient’s thyroid tumor measured 4.40 cm in the long dimension and 2.83 cm in the short dimension.

**Figure 2.**
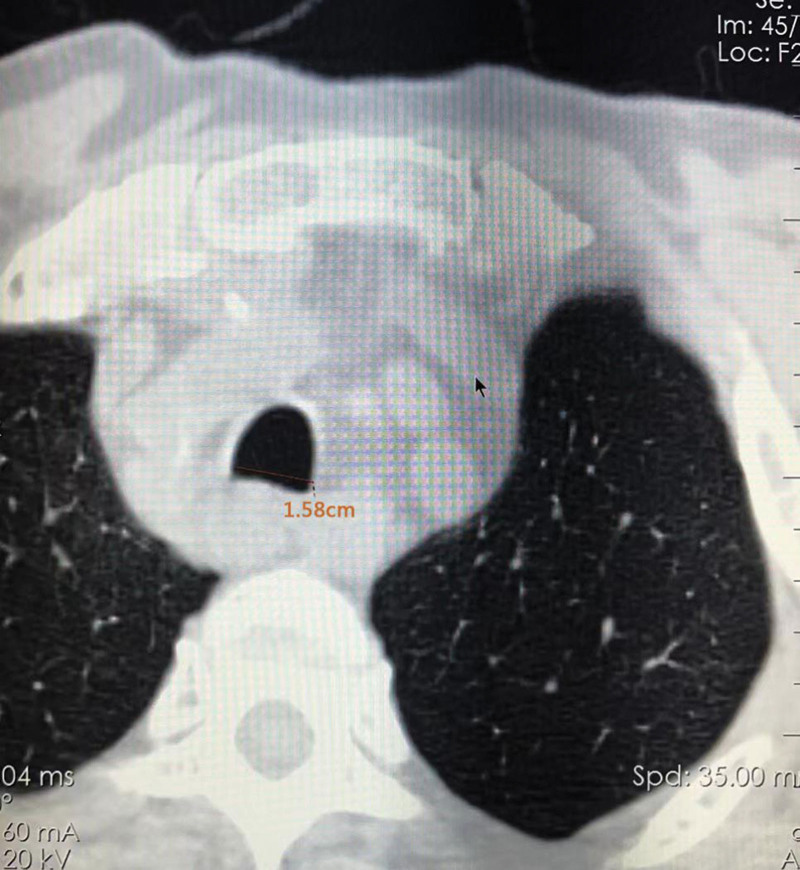
The short diameter of the patient’s normal trachea was 1.58 cm.

**Figure 3.**
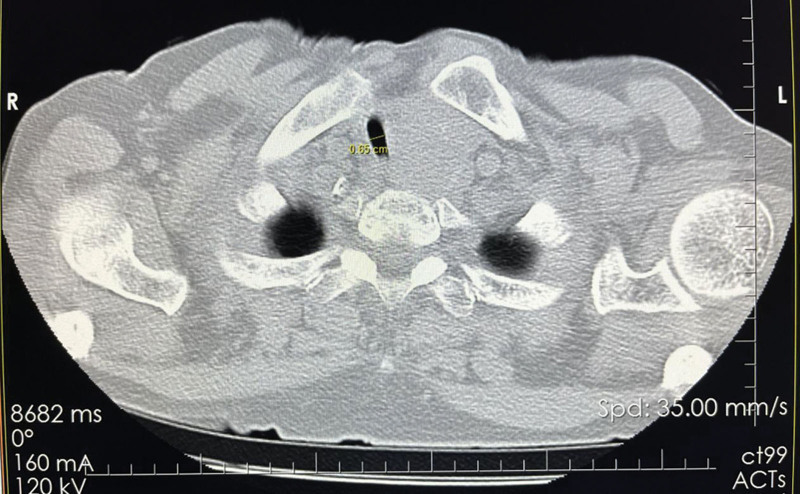
The short diameter of the patient’s tracheal stenosis was 0.65 cm.

Body weight: 63 kg T: 37.0°C HR: 78 bpm RR: 20 bpm BP: 135/69 mm Hg, clear consciousness, acute face, slight shortness of breath. The neck was soft, the trachea deviated to the right. A mass with a size of 4.40 × 2.83 cm (Fig. 1) was palpable on the left side of the neck. It was hard, ill-defined, and poorly mobile. There was local swelling and tenderness on the left side of the chest without bone rubbing. The breath sounds were coarse in both lungs, and moist rales were heard in the left lung. The muscles of the left limb were slightly weakened, but the muscle tension was normal, and the rest was normal. Arozullah score: 27 points; Cardiac function classification: grade II; Goldman score: 11 points. Evaluation of difficult airway: The LEMON law: (L) the patient does not have small mandible/large tongue/short neck; (E) 3-3-2 rule: degree of mouth opening >3 transverse fingers, chin bone-hyoid bone distance >3 transverse fingers, hyoid bone-thyroid cartilage notch distance >2 transverse fingers; (M) Modified Mallampati classification was grade I; The direct vision laryngoscopy score was grade I. (O) There was no dysphagia, stridor, or deep voice. The degree of tracheal stenosis (Cotton classification): degree I (Tracheal stenosis: [1.58–0.65]/1.58*100% = 58.86%) (Fig 2, Fig 3). (N) Range of motion of the neck was normal.

### 2.2. Laboratory examinations

Liver function and electrolytes: GLU 5.80 mmol/L, K^+^ 3.93 mmol/L, ALT 24 U/L, AST 32 U/L, total protein 60.10 g/L, albumin 35.50 g/L. Blood gas analysis: pH 7.47, PaO_2_ 45.9 mm Hg, PCO_2_ 39 mm Hg, total carbon dioxide 29.8 mmol/L, actual bicarbonate 28.65 mmol/L, respiratory index 1.44. Blood routine examinations: WBC 5.23 × 10^9^/L, RBC 4.45 × 10^12^/L, HGB 130.00 g/L, HCT 39.3%. N-terminal pro-brain natriuretic peptide 826.10 pg/mL. Blood coagulation and renal function were normal.

### 2.3. Imaging examinations

A 3-dimensional CT of the chest showed left pneumothorax, with the lung compressed by approximately 10%, and multiple rib fractures on the left side, some of which were obsolete. There were aortic and coronary calcium plaques, as well as a left pleural effusion. The left lobe of the thyroid gland was enlarged in volume with uneven density, resulting in tracheal compression.

#### 2.3.1. Preparation before anesthesia

Preparation before anesthesia: Prepare according to difficult airway guidelines^[11]^: Inform the patient of a known or suspected difficult airway; Before anesthesia and during the operation, experts from the otolaryngology head and neck surgery department were invited to the surgical bedside to prepare for tracheotomy at all times; Prepare a tracheotomy kit, direct laryngoscope, difficult intubation scope, video laryngoscope, light rod, guide wire, aspirator, sputum suction tube, oropharyngeal and nasopharyngeal airway, as well as other tracheal intubation equipment; Prepare the subglottic airway from a 3 to 6 size endotracheal tube and the supraglottic airway from a 3 to 5 size laryngeal mask. Prepare a difficult intubation emergency rescue vehicle (with all drugs needed for rescue) and defibrillator. If individual techniques encounter difficulties, combination techniques can be used.^[11]^

#### 2.3.2. Anesthesia administration

When entering the room, the patients indices were BP: 135/69 mm Hg, HR: 56 bpm, RR: 17 bpm, T: 36.5°C, S_P_O_2_: 97% during air inhalation, oxygen inhalation for mask 5 L/min, warm fan to keep warm. ECG, NIBP, pulse oximeter saturation (SpO_2_), T, RR, urine output, bispectral index (BIS) and partial pressure of end-tidal carbon dioxide (P_ET_CO_2_) were monitored. Intravenous access was opened and penehyclidine hydrochloride 0.01 mg/kg was given intramuscularly, tropisetron 5 mg was given intravenously, dexamethasone 5 mg was given intravenously and dexmedetomidine was administered using a pump at 16 µg/10 min. Epidural catheterization was performed at T7/T8, and a test dose of 5 mL 2% lidocaine was given. After 5 minutes, 8 mL 2% lidocaine was injected to make the block level reach T3–T12, after which 3 mL 2% lidocaine was added every 45 minutes (2 times in total). At the same time, a slow esketamine (ESK) infusion at a rate of 10 to 15 µg/kg/min was given over 35 minutes from the beginning of the infusion to the beginning of the operation, and the BIS value decreased from 91 to 77 (the cumulative dose of ESK was 29 mg at this time, the Ramsay score was 4). At the beginning of the operation, ESK infusion was continued, and the BIS value decreased to 73 after 15 minutes (the cumulative use of ESK was 51 mg at this time, and the patient entered the state of anesthesia). Thereafter, the BIS value was maintained at approximately 73 during the operation. Pump infusion of ESK was stopped after thoracoscopic exploration (the cumulative dose of ESK was 92 mg over 140 min). The anesthesia lasted 175 minutes and the operation lasted 139 minutes. Lactated Ringer’s solution 800 mL and succinylated gelatin 500 mL were infused during the operation. The blood loss was approximately 80 mL, and the vital signs were stable during the operation. Postoperative intravenous analgesia via a patient-controlled pump: esketamine 50 mg + flurbiprofen acetate 200 mg + dexmedetomidine 75 µg + tropisetron 5 mg + 0.9% NaCl to 100 mL, pump speed 2 mL/h, bolus 0.5 mL/15 min. At the end of the operation, S_P_O_2_ was maintained above 95% during mask oxygen inhalation. The patient could respond to the call, move his limbs independently, and breathe deeply. Six minutes after the end of anesthesia, the patient was transferred back to the ward, with continuous monitoring and mask oxygen inhalation.

#### 2.3.3. Physical examination in the ward

T: 36.3°C, P: 62 bpm, RR: 19 bpm, BP: 128/72 mm Hg, SpO_2_: 97%, visual analogue scale: 2 points, bilateral pupils equal and round, light reflex positive. The breath sounds of both lungs were coarse, and no dry or wet rales were heard. The heart rhythm was consistent, and no pathological murmur was heard in the auscultation area of each valve. The patient was able to move freely after surgery and could eat normally after 6 hours. The postoperative visual analogue scale score was approximately 2 points, and the follow-up was unremarkable. Written informed consent was obtained from the patient’s son for the publication of this case report.

## 3. Discussion

The difficulties of anesthesia in this case were as follows: The patient was older and had given up the treatment of a thyroid tumor, so it was impossible to relieve or tracheal compression in the short or long term; Although the shortest diameter of the trachea was 0.65 cm according to imaging, there was no guarantee of successful smooth insertion of the corresponding endotracheal tube; Although the imaging evaluation showed that the thyroid tumor did not invade the inner wall of the trachea, it was not possible to determine whether the blood vessels of the compressed inner wall of the trachea were proliferating. If the tracheal tube were to damage the infiltrating vascular tissue, even a short period of massive bleeding would lead to asphyxia, with disastrous consequences; After applying muscle relaxants during general anesthesia induction, the loss of support around the thyroid tumor may aggravate tracheal stenosis, resulting in insufficient oxygen supply or asphyxia; Even if all goes well and the patient is intubated successfully, it would be unclear when the endotracheal tube can be removed after the operation. Moreover, a possible tracheal collapse after removal of the endotracheal tube is also a challenge; The location of the thyroid tumor was very low, so that the narrowest tracheal segment was 3 cm below the cricoid cartilage and 1.5 cm above the jugular notch, at the level of the second thoracic vertebra. Therefore, preoperative tracheotomy is risky and difficult.

Opioids are associated with multiple acute side effects, including nausea, pruritus, respiratory depression, and constipation.^[12]^ When inappropriately treated, acute pain transforms into chronic pain, and the opioid epidemic has become a major public health problem, driving up healthcare costs and reducing patient satisfaction.^[13]^ OFA is a multimodal pain control technique that uses non-opioid drugs to provide adequate analgesia during the perioperative period.^[14]^ One study showed that OFA-based on intravenous-inhalation general anesthesia combined with thoracic paravertebral nerve block may improve postoperative recovery in patients undergoing video-assisted thoracoscopic surgery.^[15]^ A randomized controlled study evaluated changes in pain threshold index and found that OFA combined with dexmedetomidine was feasible for intraoperative pain management.^[16]^ Dexmedetomidine has anxiolytic, sedative, analgesic, saliva-reducing and other effects. As an adjuvant, dexmedetomidine can inhibit the side effects of ketamine such as tachycardia and hypertension.^[17]^ Preclinical experiments have shown that intravenous infusion of dexmedetomidine in combination with ketamine reduces hyperactivity of brain neurons and alleviates ketamine-induced psychiatric side effects.^[18]^

A large number of studies comparing intubated and non-intubated general anesthesia, especially in Asian countries, have shown that non-intubated thoracoscopic surgery can reduce the incidence of postoperative complications, shorten the length of hospital stay, and reduce perioperative mortality, indicating that non-intubated thoracoscopic surgery is a safe, effective and feasible anesthesia technique for thoracic surgery.^[19]^ The necessary local anesthesia for non-intubation thoracoscopic surgery includes thoracic epidural anesthesia, cervical epidural anesthesia, paravertebral block, intercostal nerve block, local anesthesia at the incision site, local vagus nerve block, erector spinae plane block, serratus anterior plane block and pectoralis major nerve block.^[20]^ Non-intubated thoracoscopic surgery can increase the incidence of hypercapnia and intraoperative cerebral oxygen saturation, reduce systemic inflammation, promote early postoperative activities, and accelerate the postoperative recovery of patients.^[21]^ This is also demonstrated in the present patient, who was returned to the ward 6 minutes after the end of surgery. Notably, he was able to move freely after surgery and eat normally 6 hours later. Pulse oximetry reflects oxygenation, and a decreases in SpO_2_ values can occur long after hypoventilation but is not detected.^[22]^ In this case, P_ET_CO_2_ was monitored during the operation and maintained at about 27 mm Hg. Although this value cannot be the true end-breath carbon dioxide concentration of patients, because P_ET_CO_2_ is timely, the coherence and regularity of its waveform can help anesthesiologist determine the incidence and degree of respiratory depression. Changes in P_ET_CO_2_ seem to precede changes in SpO_2_, and periods of apnea, decreased respiratory rate, and hypoventilation are easily detected by carbon dioxide monitoring. By measuring P_ET_CO_2_, it can provide critical and timely information on the ventilatory status of patients, giving early warning of hazards.^[23–25]^

Compared with opioid anesthesia, the OFA protocol achieved an equally effective intraoperative pain threshold index in thoracoscopic surgery.^[16]^ Substitution of opioids with ESK can reduce the incidence of mild chronic postoperative pain and side effects in patients undergoing thoracoscopic surgery.^[26]^ In a 70 kg patient, 12 to 24 mg/h (5.7–11.5 µg/kg/min) of ESK is effective in relieving remifentanil-induced respiratory depression (mainly by increasing the respiratory rate).^[27]^ In this case, ESK was pumped at a dose of 10 to 15 µg/kg/min, similar to the published dose, and respiratory depression did not occur. ESK has effective anesthetic and analgesic effects, while still allowing spontaneous breathing and airway reflexes.^[28]^ In addition, it can antagonize N-methyl-D-aspartic acid receptors, relax bronchial muscle activity, inhibit histamine-induced bronchial contraction, as well as reduce tracheal and bronchial muscle spasms.^[29]^ESK is effective and neuroprotective when used for the swallowing reflex, eye blink reflex, cough reflex and vomiting reflex during anesthesia.^[7]^ In this case, the BIS value was monitored during the operation, although it could not completely reflect the depth of anesthesia. A comparison of the BIS values before and after anesthesia can also be used to roughly judge the depth of anesthesia and guide the pump injection rate of ESK. It has been shown that intravenous ketamine administration under general anesthesia affects BIS values in children in a dose-dependent manner.^[30]^

It is important to note that intrathoracic vagus nerve block and topical anesthesia with 3 to 5 mL lidocaine on the surface of the lung must be performed according to the existing protocol for thoracotomy without intubation.^[31]^ To avoid the hering-breuer reflex caused by intraoperative exploration and compression of the lung, the SpO_2_ temporarily decreased below 60%.

In conclusion, the patient presented here had stable vital signs, perfect intraoperative analgesia, and rapid postoperative recovery, so that he could be directly returned to the ward after 6 minutes. Although SpO_2_ transiently decreased below 60% during thoracoscopic exploration, the OFA strategy may be safe and effective for enhanced recovery after surgery in patients with tracheal stenosis undergoing video-assisted thoracoscopic surgery.

## Author contributions

**Project administration:** Jingwei Dai, Mengya Yang, Hefei Xu.

**Software:** Danmin Wang, Zhaoqiang Zeng.

**Supervision:** Xiaobo Yu, Jingwei Dai.

**Writing – original draft:** Mengya Yang.

**Writing – review & editing:** Xia Xu, Jingwei Dai.

## References

[1] Buckingham W Beatty AJ Brasher CA. The technique of administering epidural anesthesia in thoracic surgery. Dis Chest. 2009;136:561. 10.1378/chest.17.5.561 15411863

[2] Angst MS Clark JD. Opioid-induced hyperalgesia: a qualitative systematic review. Anesthesiology. 2006;104:570–87. 16508405 10.1097/00000542-200603000-00025

[3] Lee M Silverman SM Hansen H. Comprehensive review of opioid-induced hyperalgesia. Pain Physician. 2011;14:145–61. 21412369

[4] Practice guidelines for the prevention, detection, and management of respiratory depression associated with neuraxial opioid administration: an updated report by the American society of anesthesiologists task force on neuraxial opioids and the American society of regional anesthesia and pain medicine. Anesthesiology. 2016;124:535–52. 26655725 10.1097/ALN.0000000000000975

[5] Bello M Oger S Bedon-Carte S. Effect of opioid-free anaesthesia on postoperative epidural ropivacaine requirement after thoracic surgery: a retrospective unmatched case-control study. Anaesth Crit Care Pain Med. 2019;38:499–505. 30731138 10.1016/j.accpm.2019.01.013

[6] Selim J Jarlier X Clavier T. Impact of opioid-free anesthesia after video-assisted thoracic surgery: a propensity score study. Ann Thorac Surg. 2022;114:218–24. 34662540 10.1016/j.athoracsur.2021.09.014

[7] Trimmel H Helbok R Staudinger T. S(+)-ketamine: current trends in emergency and intensive care medicine. Wien Klin Wochenschr. 2018;130:356–66. 29322377 10.1007/s00508-017-1299-3 PMC6061669

[8] Batchelor T Rasburn NJ Abdelnour-Berchtold E. Guidelines for enhanced recovery after lung surgery: recommendations of the enhanced recovery after surgery [ERAS(R)] society and the European society of thoracic surgeons (ESTS). Eur J Cardiothorac Surg. 2019;55:91–115. 30304509 10.1093/ejcts/ezy301

[9] Brunelli A Thomas C Dinesh P. Enhanced recovery pathway versus standard care in patients undergoing video-assisted thoracoscopic lobectomy. J Thorac Cardiovasc Surg. 2017;154:2084–90. 28728783 10.1016/j.jtcvs.2017.06.037

[10] Martin LW Sarosiek BM Harrison MA. Implementing a thoracic enhanced recovery program: lessons learned in the first year. Ann Thorac Surg. 2018;105:1597–604. 29510097 10.1016/j.athoracsur.2018.01.080

[11] Apfelbaum JL Hagberg CA Connis RT. 2022 American society of anesthesiologists practice guidelines for management of the difficult airway. Anesthesiology. 2022;136:31–81. 34762729 10.1097/ALN.0000000000004002

[12] Frauenknecht J Kirkham KR Jacot-Guillarmod A. Analgesic impact of intra-operative opioids vs. opioid-free anaesthesia: a systematic review and meta-analysis. Anaesthesia. 2019;74:651–62. 30802933 10.1111/anae.14582

[13] Feizerfan A Sheh G. Transition from acute to chronic pain. Contin Educ Anaesth Crit Care Pain. 2015;15:98–102.

[14] Lavand’homme P Estebe JP. Opioid-free anesthesia: a different regard to anesthesia practice. Curr Opin Anaesthesiol. 2018;31:556–61. 29994942 10.1097/ACO.0000000000000632

[15] Wang XR Jia XY Jiang YY. Opioid-free anesthesia for postoperative recovery after video-assisted thoracic surgery: a prospective, randomized controlled trial. Front Surg. 2023;9:1035972. 36684254 10.3389/fsurg.2022.1035972 PMC9852053

[16] An G Zhang Y Chen N. Opioid-free anesthesia compared to opioid anesthesia for lung cancer patients undergoing video-assisted thoracoscopic surgery: a randomized controlled study. PLoS One. 2021;16:e0257279. 34555043 10.1371/journal.pone.0257279 PMC8460000

[17] Kim JG Lee HB Jeon SB. Combination of dexmedetomidine and ketamine for magnetic resonance imaging sedation. Front Neurol. 2019;10:416. 31105637 10.3389/fneur.2019.00416 PMC6492498

[18] Chu Q Zhu K Bai Y. A single low dose of dexmedetomidine efficiently attenuates esketamine-induced overactive behaviors and neuronal hyperactivities in mice. Front Hum Neurosci. 2021;15:735569. 34712126 10.3389/fnhum.2021.735569 PMC8545873

[19] Janík M Juhos P Lučenič M. Non-intubated thoracoscopic surgery-pros and cons. Front Surg. 2021;8:801718. 34938770 10.3389/fsurg.2021.801718 PMC8687085

[20] Grott M Eichhorn M Eichhorn F. Thoracic surgery in the non-intubated spontaneously breathing patient. Respir Res. 2022;23:379. 36575519 10.1186/s12931-022-02250-z PMC9793515

[21] Huang Y Bo Y Li Y. The impact of tubeless anesthesia versus intubated anesthesia on cerebral oxygen saturation and postoperative cognitive function in patients undergoing video-assisted thoracoscopic surgery: a randomized trial. J Thorac Dis. 2022;14:4012–30. 36389295 10.21037/jtd-22-1165 PMC9641321

[22] Zhang X Kassem MA Zhou Y. A brief review of non-invasive monitoring of respiratory condition for extubated patients with or at risk for obstructive sleep apnea after surgery. Front Med (Lausanne). 2017;4:26. 28337439 10.3389/fmed.2017.00026 PMC5340767

[23] Franchi LM Maggi JC Nussbaum E. Continuous end-tidal CO2 in pediatric bronchoscopy. Pediatr Pulmonol. 1993;16:153–7. 8309738 10.1002/ppul.1950160303

[24] Odom-Forren J. Capnography and sedation: a global initiative. J Perianesth Nurs. 2011;26:221–4. 21803269 10.1016/j.jopan.2011.07.001

[25] McNeill MM Hardy Tabet C. The effectiveness of capnography versus pulse oximetry in detecting respiratory adverse events in the Postanesthesia Care Unit (PACU): a narrative review and synthesis. J Perianesth Nurs. 2022;37:264–269.e1. 34974968 10.1016/j.jopan.2021.03.013

[26] Yan H Chen W Chen Y. Opioid-free versus opioid-based anesthesia on postoperative pain after thoracoscopic surgery: the use of intravenous and epidural esketamine. Anesth Analg. 2023;137:399–408. 37267129 10.1213/ANE.0000000000006547

[27] Jonkman K van Rijnsoever E Olofsen E. Esketamine counters opioid-induced respiratory depression. Br J Anaesth. 2018;120:1117–27. 29661389 10.1016/j.bja.2018.02.021

[28] Xin N Yan W Jin S. Efficacy of analgesic propofol/esketamine and propofol/fentanyl for painless induced abortion: a randomized clinical trial. Biomed Res Int. 2022;2022:5095282. 35722469 10.1155/2022/5095282 PMC9203225

[29] Lu X Tang L Lan H. A comparison of intranasal dexmedetomidine, esketamine or a dexmedetomidine-esketamine combination for induction of anaesthesia in children: a randomized controlled double-blind trial. Front Pharmacol. 2021;12:808930. 35185548 10.3389/fphar.2021.808930 PMC8848099

[30] Peltoniemi MA Hagelberg NM Olkkola KT. Ketamine: a review of clinical pharmacokinetics and pharmacodynamics in anesthesia and pain therapy. Clin Pharmacokinet. 2016;55:1059–77. 27028535 10.1007/s40262-016-0383-6

[31] Lan L Cen Y Zhang C. A propensity score-matched analysis for non-intubated thoracic surgery. Med Sci Monit. 2018;24:8081–7. 30415268 10.12659/MSM.910605 PMC6410560

